# Case Report: A 62-Year-Old Woman With Contrast-Induced Encephalopathy Caused by Embolization of Intracranial Aneurysm

**DOI:** 10.3389/fsurg.2021.689713

**Published:** 2021-07-19

**Authors:** Ying Zhang, Ming Zhou, Dong Wang, Tao Liu, Pengfei Chang, Jie Zhang, Rui Zhang, Yumin Luo, Ping Liu

**Affiliations:** ^1^Department of Encephalopathy, Traditional Chinese Medicine Hospital of Weifang, Weifang, China; ^2^Department of Neurology, Xuanwu Hospital of Capital Medical University, Beijing, China

**Keywords:** contrast-induced encephalopathy, complication of endovascular treatment, aneurysm embolization, computer tomography, cerebral edema

## Abstract

Contrast-induced encephalopathy (CIE) is a rare complication of endovascular treatment and is extensively reported as a transient and reversible phenomenon. This report describes a 62-year-old woman for embolization of an internal carotid artery (ICA) aneurysm. The operation was successful, but postoperation the patient suffered unconsciousness, blindness, hemiplegia, ophthalmoplegia, fever, and seizures. CT of the brain without the contrast showed widespread edema in the right cerebral hemisphere, which is involved in the frontal, parietal, temporal, and occipital lobes. She was diagnosed with CIE in time and treated with supportive management as soon as possible, and fortunately, the patient improved a benign course and was discharged without any neurological deficits. This study emphasizes the prevention of the CIE and the importance of early diagnosis and symptomatic treatment.

## Introduction

Contrast-induced encephalopathy (CIE) is a rare complication of angiography. The first case of CIE was described in 1970 as transient cortical blindness after coronary angiography (1). Clinical manifestations include encephalopathy, seizures, cortical blindness, and focal neurological deficits, such as ophthalmoplegia (2). Herein, a case of CIE following embolization of intracranial aneurysm is described, in which the severe neurological deficits were reversed.

## Case Report

**Figure 1:**
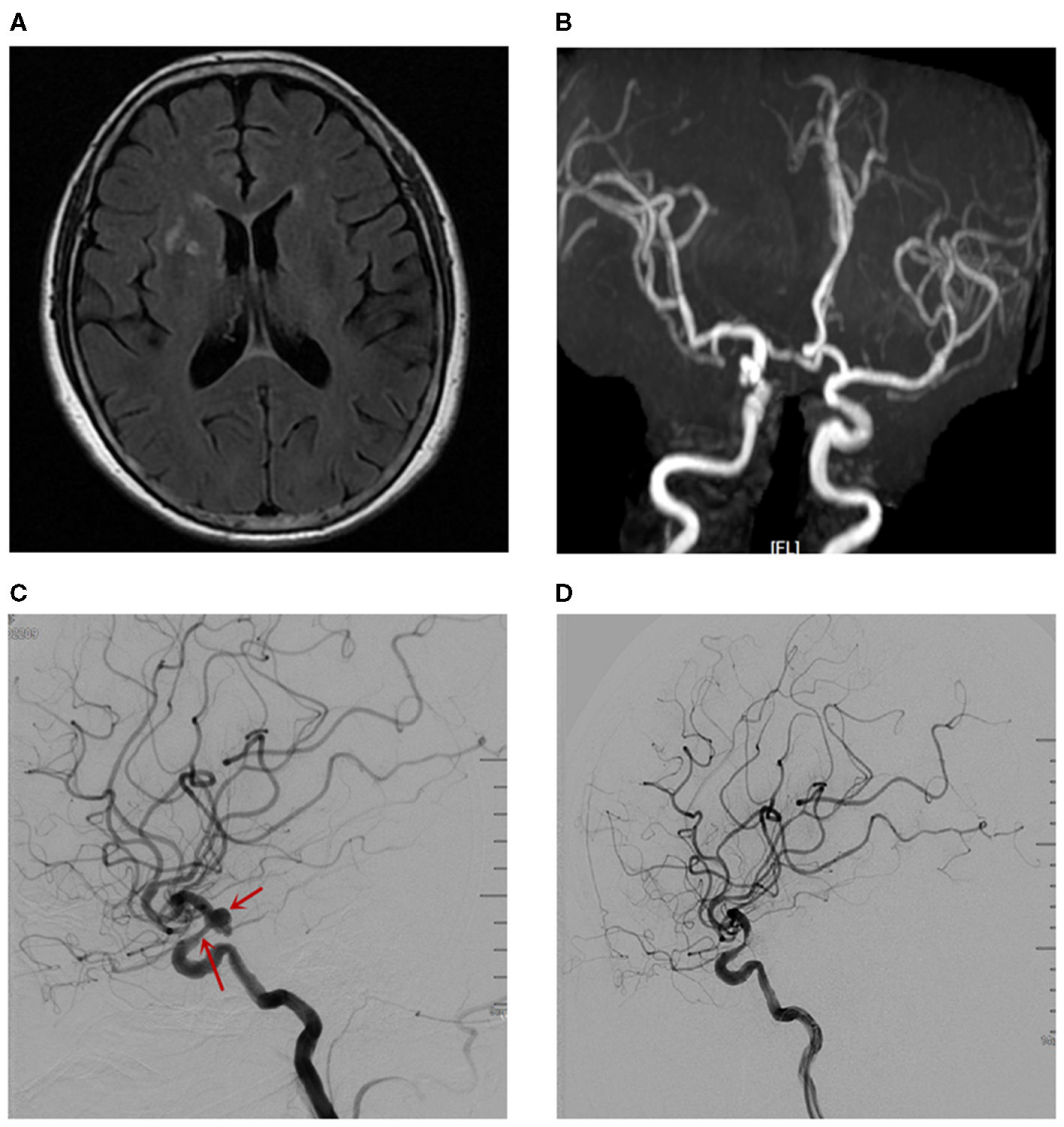
The imaging examinations before CIE. **(A)** MRI of the brain. **(B)** MRA detected the aneurysm in the communicating segment of the right ICA. **(C)** The DSA revealed an aneurysm with size of 7.4mm*6.1mm in the right ICA-communicating segment, and mild stenosis in right ICA-ophtalmic segment. **(D)** The DSA revealed a coiling embolization of the aneurysm.

A 62-year-old woman with a history of hypertension was admitted to our hospital due to headache for 20 days. On physical examination, blepharoptosis was found in the right eye. MRI of the brain detected changes in signals that consist of multiple lacunar infarctions in the right basal ganglia and bilateral demyelination in the centrum semiovale (Figure 1A). Magnetic resonance angiography (MRA) of the brain indicated an aneurysm in the communicating segment of the right internal carotid artery (ICA) (Figure 1B). Then, the patient underwent digital silhouette angiography

(DSA) for a further assessment, with the total amount of 200 ml iodixanol (iodine concentration 270 mgi/ml; osmotic pressure 290 mOsm/kg H_2_O, Yangtze River Pharm, China). The DSA revealed an aneurysm with a size of 7.4 mm ^*^ 6.1 mm in the right communicating segment of ICA, and mild stenosis in right ophthalmic segment of ICA (Figure 1C). There was no complication during and after the operation.

**Figure 2:**
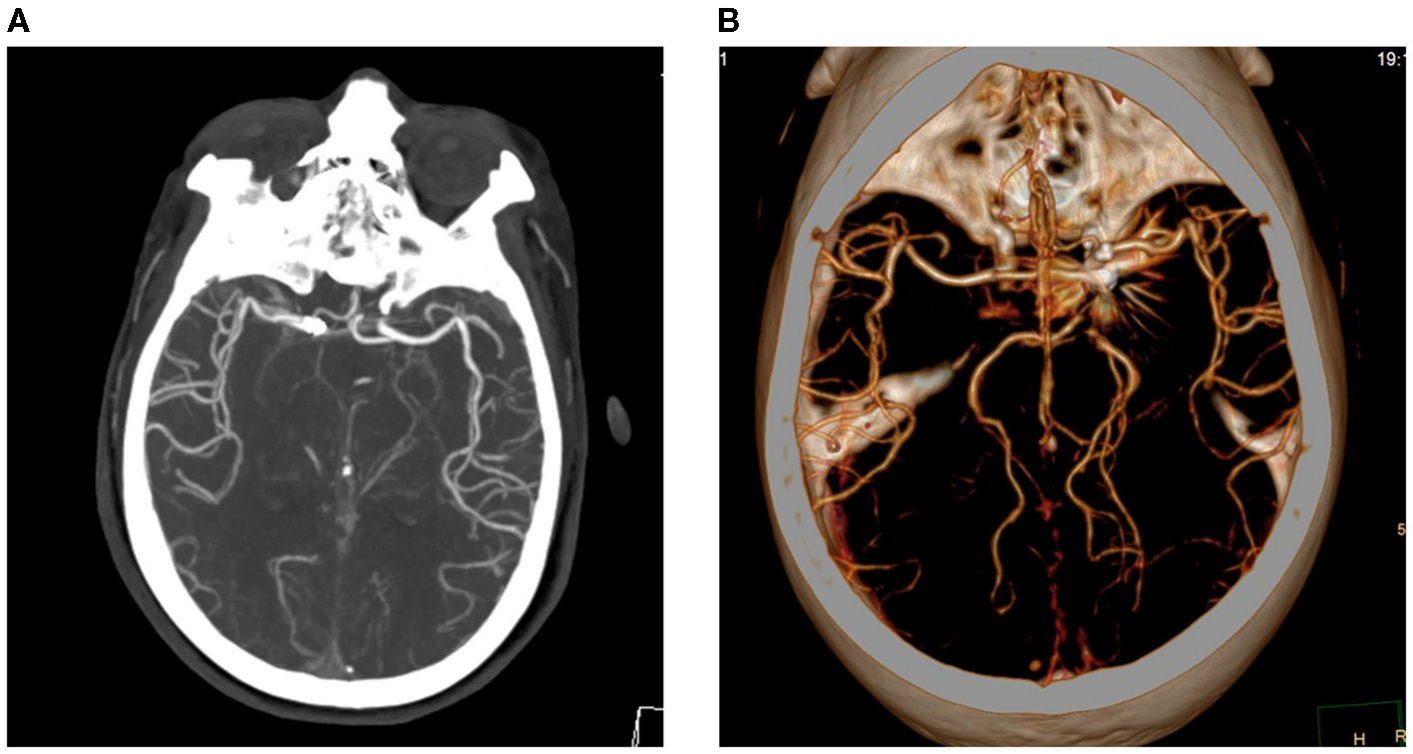
CTA performed 2 hours later indicated no occlusion of the right ICA **(A,B)**.

**Figure 3:**
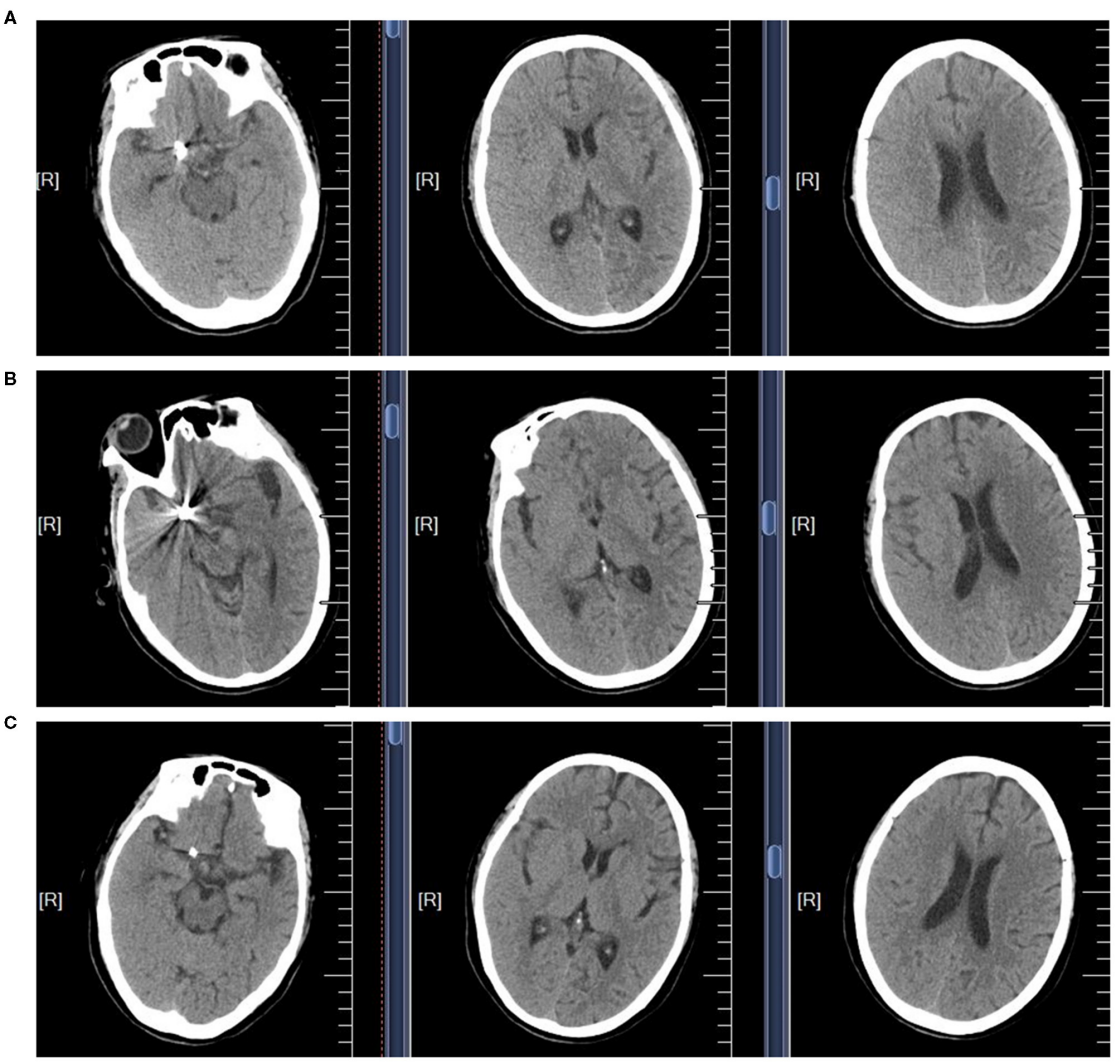
The non-contrast CT scans of the brain in CIE patient. **(A)** The brain CT scan performed immediately after the operation. **(B)** The brain CT scan performed in the following day. **(C)** The brain CT scan performed in the third day.

The patient received oral aspirin (100 mg/day) and clopidogrel (75 mg/day) for 5 days, after which a coiling embolization of the aneurysm was performed under general anesthesia. She received successful coiling of the aneurysm using two stent remodeling techniques (Figure 1D). Postcoiling angiography showed completed occlusion of the aneurysm, and all the arteries were shown to be patent in the final angiogram. The total amount of iodixanol (iodine concentration 270 mgi/ml; osmotic pressure 290 mOsm/kg H_2_O, Yangtze River Pharm, China) administrated during this procedure was also 200 ml, and the systolic blood pressure (SBP) was maintained at about 110 mmHg during the intraoperative period. About 10 min after the operation, the patient woke up, but she became confused and suffered blindness, and the eyes stared to the right side. The diameters of the pupils were 3 mm bilaterally with light reflex sensitivity. One hour later, the weakness in the left limb decreased (regained 4/5 strength), and the SBP gradually increased to about 180 mmHg. A non-contrast CT of the brain performed immediately after the operation showed edema in the right cerebral hemisphere and involved the frontal, parietal, temporal, and occipital lobes, and there was no hemorrhage (Figure 3A). CT angiography (CTA) performed 2 h later indicated no occlusion of the right ICA (Figures 2A,B). She was diagnosed with CIE based on the acute onset of symptoms after cerebral angiography and distinctive CT findings. We initiated hydration therapy for the excretion of the contrast agent and used urapidil to control SBP at about 140 mmHg; meanwhile, mannitol was used to improve the cerebral edema. On the following day, the patient had a fever up to 38.5°C, weakness in the left limb decreased (regained 0/5 strength), and suffered generalized tonic-clonic seizures for twice. CT reviewed persistent edema in the right cerebral hemisphere (Figure 3B). We applied methylprednisolone pulse 80 mg/day for CIE and diazepam 10 mg/day for symptomatic epilepsy. Fortunately, her clinical manifestations disappeared gradually on the third day and the follow-up brain CT showed resolution of the cerebral edema (Figure 3C).

## Discussion

This case report demonstrated the following points: First, minimization of the amount of contrast medium and hydration may prevent the CIE. Second, early diagnosis, active blood pressure control, and symptomatic treatment are important to reduce the incidence of CIE and alleviate its disability. CIE is a very rare complication of endovascular interventions. The incidence of CIE seems to be lower in recent years due to the increased use of nonionic contrast agents. Though neurological status usually occurs self-limiting over a period of days (3), there are persistent neurological deficits and irreversible fatal cerebral edema cases reported (4, 5). Therefore, CIE should be kept in mind as a complication of angiography and supportive therapy should be initiated as soon as possible.

The mechanism and cause of CIE are associated with the disruption of the blood-brain barrier (BBB). Contrast agents can damage the BBB and facilitate its own entry into the brain, which is considered to be one of the mechanisms of CIE (4). Studies have been indicated that the risk factors of CIE included hypertension, renal disorder, acute cerebral infarction, the low temperature of contrast media, and endovascular surgery requiring a higher dose of contrast agents (6). In our case, though the total amount of contrast agents is not excessive, the contrast agent was injected repeatedly into a single vessel, and the cumulative injection may disrupt the BBB. Meanwhile, the case presented with lacunar infarction and white matter lesion indicated small vessel disease with a certain degree of vascular endothelial damage, and the damaged BBB was vulnerable to be disrupted by the repeated injection of contrast agents into the territory of ICA with endothelial damage induced by small vessel disease.

The diagnosis of CIE is not easy, as its presentation may resemble embolism and hemorrhagic complications following angiography and percutaneous interventions. The typical radiological findings, including abnormal cortical contrast enhancement and cerebral edema, subarachnoid contrast enhancement, and striatal contrast enhancement, could help us to differentiate CIE from hemorrhage or infarction. Measuring the Hounsfield units (HU) can help differentiate subarachnoid hemorrhage (SAH) from CIE, as blood usually measures at 30–45 HU, and contrast usually measures at 80–160 HU (7). On MRI, gyral swelling and hyperintensity on T2 fluid-attenuated inversion recovery (FLAIR) image and diffusion-weighted imaging (DWI), not accompanied by changes on apparent diffusion coefficient (ADC), have been described in the previous report (8). In this case, we diagnosed CIE by the acute onset of symptoms after cerebral angiography, typical CT and CTA findings, and rapid improvement in clinical and imaging findings.

There is no definitive treatment for CIE, and hydration and close observation of the patient in the postprocedural period are recommended (6). Steroids are also commonly used to improve cerebral edema by stabilizing the BBB (9). Mannitol is another type of drug used to relieve cerebral edema; however, it is because that mannitol opens the BBB and conversely induces contrast medium into the brain tissue (10). Hemodialysis is useful for the removal of the contrast medium from the blood, with approximately 80% of the contrast medium having been removed within 4 h, and there have been cases where symptoms were improved by hemodialysis (11). It is also reported the deterioration of neurological symptoms caused by cerebral edema is attributed to an osmotic gradient between the brain and the blood as a result of rapid removal of urea by hemodialysis (10, 11). For fatal cerebral edema cases, craniectomy should be considered (12), while further research studies are needed to develop the evidence-based treatment for CIE.

Though the rare incidence of CIE renders their prevention, which may help minimize the severe complication very difficult, following measures should be considered during the perioperative period. First, it is reported that CIE is an idiosyncratic response to a small dose of contrast agent, a variety of preoperative tests for contrast agents should be performed, and it is generally accepted that antiallergic drugs or corticosteroids should be used for patients who were allergic to contrast media (13). Second, hydration should be sufficient to accelerate the excretion of contrast medium during and after the operation, as long as the cardiac function is tolerated. Third, it is reported that BBB is affected by the injection of high concentrations, low temperatures, and repeated injections with short interval into the same blood vessel (14); therefore, during the procedure we should minimize the amount of contrast medium while keeping the image clear and pay attention to the details mentioned above. Fourth, with regard to hypertension, it is thought that the BBB is affected by a decline in the autoregulation blood vessels (15). We should control blood pressure in a reasonable range and avoid fluctuation of blood pressure during the whole perioperative period. Recent research studies display that improving contrast media using nanocarriers, such as nanoparticles, liposomes, micelles, nanobodies, and quantum dots, may enhance its tolerability and bioavailability because they leverage on the bioinert potential of their coating agents and also decrease the risk of CIE (16, 17).

In conclusion, we emphasize the prevention of the CIE, as well as the importance of early diagnosis and symptomatic treatment, in order to reduce the incidence of CIE and alleviate its disability. However, in the clinic, it needs experienced doctors to judge CIE quickly It may be more reasonable to extend the interval between two injections of contrast agents.

## Data Availability Statement

The original contributions presented in the study are included in the article/supplementary material, further inquiries can be directed to the corresponding author/s.

## Ethics Statement

Written informed consent was obtained from the individual(s) for the publication of any potentially identifiable images or data included in this article.

## Author Contributions

YZ, MZ, DW, TL, PC, JZ, and RZ contributed to the search and assessment of the available literature. YZ and PL wrote the manuscript. YL and PL helped revise the text to the final form. All authors contributed to the article and approved the submitted version.

## Conflict of Interest

The authors declare that the research was conducted in the absence of any commercial or financial relationships that could be construed as a potential conflict of interest.
